# Complete Thoracoscopic Sublobar Resection for Intralobar Pulmonary Sequestration Using Indocyanine Green Fluorescence: A Case Report with Literature Review

**DOI:** 10.70352/scrj.cr.25-0816

**Published:** 2026-04-08

**Authors:** Kaoru Kondo, Hidetoshi Inokawa, Masanori Okada, Riki Okita, Eiji Ikeda

**Affiliations:** 1 Division of Thoracic Surgery, National Hospital Organization Yamaguchi Ube Medical Center, Ube, Yamaguchi, Japan; 2 Department of Pathology, National Hospital Organization Yamaguchi Ube Medical Center, Ube, Yamaguchi, Japan

**Keywords:** pulmonary sequestration, indocyanine green, thoracoscopy, segmentectomy, fluorescence imaging

## Abstract

**INTRODUCTION:**

Intralobar pulmonary sequestration (IPS) is a congenital pulmonary anomaly supplied by an aberrant systemic artery. Although surgical resection is the standard treatment, complete thoracoscopic lung-preserving (sublobar) resection can be technically demanding because the border between normal and sequestrated lung may be unclear, particularly in the presence of inflammation or pleural adhesions. Indocyanine green (ICG) fluorescence imaging provides real-time perfusion-based demarcation without requiring lung inflation. We present a case of IPS treated by complete thoracoscopic sublobar resection under ICG guidance and review the literature to clarify the role of ICG in complete thoracoscopic lung-preserving surgery for IPS.

**CASE PRESENTATION:**

A 21-year-old man was referred after an abnormal opacity was detected on routine chest radiography. CT demonstrated a mass in the right lower lobe with an enhancing intralesional vessel. 3D-CT identified an aberrant systemic artery arising from the abdominal aorta with venous drainage into the basal pulmonary vein, consistent with IPS (Pryce type III). Thoracoscopy revealed multiple string-like pleural adhesions requiring adhesiolysis to obtain an adequate operative view. After proximal control of the aberrant systemic artery and division of the draining vein, intravenous ICG (10 mg) was administered. Fluorescence imaging clearly delineated the border between perfused normal lung and the non-perfused sequestrated area, enabling complete thoracoscopic lung-preserving resection along the demarcation line. The postoperative course was uneventful; the chest drain was removed on POD 1, and the patient was discharged on POD 9. Follow-up CT at 6 months showed no residual lesion.

**CONCLUSIONS:**

This case and literature review suggest that ICG fluorescence is a practical adjunct for defining the resection line in IPS and may help maintain a complete thoracoscopic lung-preserving approach, including in selected patients with pleural adhesions, when careful patient selection and secure vascular control are ensured.

## Abbreviations

HFJV
: high-frequency jet ventilation
ICG
: indocyanine green
IPS
: intralobar pulmonary sequestration
ISP
: intersegmental plane
RATS
: robotic-assisted thoracic surgery
VATS
: video-assisted thoracic surgery

## INTRODUCTION

IPS is a congenital pulmonary malformation that may lead to infectious and other complications; therefore, surgical resection is considered the standard treatment.^1)^ Historically, lobectomy has frequently been selected, and open thoracotomy has remained one of the common approaches in adult surgical series (lobectomy 80.6%; open thoracotomy 37.1%).^1)^ With advances in preoperative imaging and minimally invasive approaches, sublobar resection (segmentectomy or wedge resection) has also been performed in selected patients in whom preservation of normal lung parenchyma is desirable.^1)^

A major challenge in sublobar resection for IPS is the accurate identification of the resection line between normal lung and sequestrated lung tissue. Various techniques have been reported to delineate the ISP, including inflation–deflation, selective segmental inflation, and several image-guided methods.^2)^ Among these, intravenous ICG fluorescence is widely used in anatomical segmentectomy because it enables real-time visualization without lung inflation and provides a stable operative field.^2)^

ICG fluorescence has also been applied to pulmonary sequestration surgery. In a recent IPS series, the use of ICG was associated with increased selection of minimally invasive approaches (VATS 83% in the ICG group vs. 0% in the non-ICG group) and a higher rate of segmentectomy (100% vs. 46%).^3)^ Meanwhile, surgeons may consider thoracotomy when intense adhesions are anticipated due to prior inflammation, and lobectomy may be selected when the lesion is extensive.^3)^ In addition, inadequate fluorescence visualization (“poor staining”) has been reported under certain conditions (e.g., obstructive pulmonary disease), underscoring the importance of contingency planning.^4)^ Reports focusing on ICG-guided thoracoscopic sublobar resection in IPS complicated by intrathoracic adhesions remain limited.

Here, we report a case of right lower lobe IPS with a history of recurrent pneumonia in which a complete thoracoscopic sublobar resection was successfully completed with the aid of intravenous ICG fluorescence to confirm the resection line. We also review the literature regarding methods for resection-line/ISP identification and discuss the positioning of ICG fluorescence in IPS with inflammatory adhesions.

## CASE PRESENTATION

**Fig. 1:**
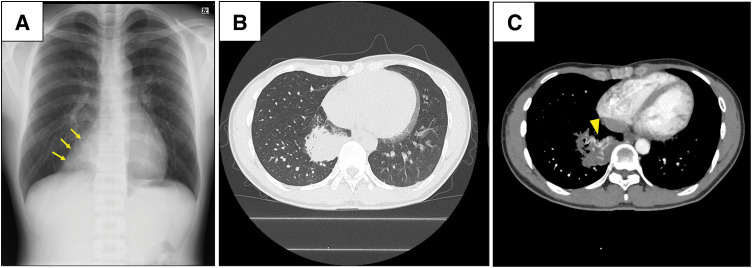
Preoperative imaging findings. (**A**) Chest radiograph obtained during a routine health check showing a well-circumscribed opacity in the medial aspect of the right lower lobe (arrows). (**B**) Contrast-enhanced CT demonstrating a mass in the right lower lobe. (**C**) Contrast-enhanced CT showing an enhanced vessel-like structure within the mass (arrowhead), suggesting a vascular lesion.

**Fig. 2:**
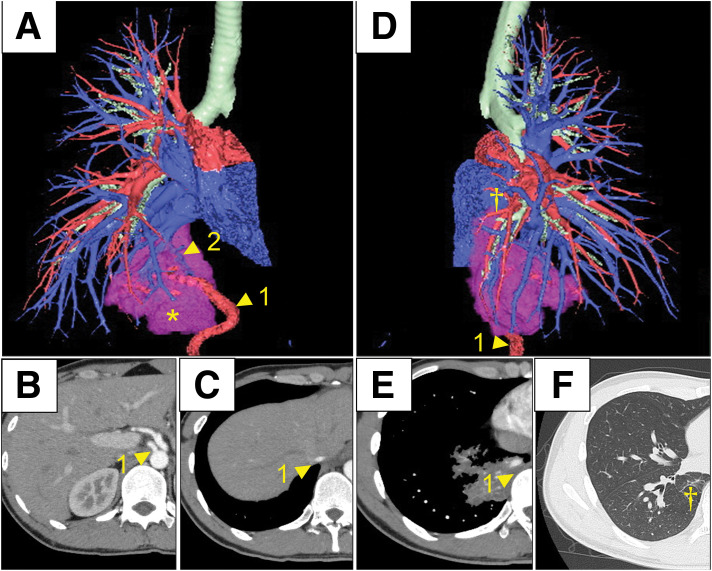
3D-CT findings. (**A**) 3D-CT (anterolateral view): arrowhead 1, an aberrant systemic artery arising from the abdominal aorta and supplying the lesion; arrowhead 2, venous drainage into the basal pulmonary vein; *, the sequestered area. (**B**, **C**) Axial contrast-enhanced CT demonstrating a tubular enhancing vessel (1) coursing from the abdominal aorta toward the lesion. (**D**) 3D-CT (posterior view): †, no identifiable segmental bronchus entering the lesion, consistent with sequestration. (**E**) Axial lung-window CT showing an intralesional aberrant systemic artery (1). (**F**) Axial lung-window CT confirming †, absence of a bronchus corresponding to B10 and lack of communication with the tracheobronchial tree. *, sequestered area; †, absence of a segmental bronchus entering the lesion

A 21-year-old man with a history of pneumonia and meningitis was referred to our hospital because a chest radiograph obtained during a health checkup revealed an abnormal shadow (**Fig. 1A**). Chest CT showed a mass in the right lower lobe (**Fig. 1B**) containing an enhanced vessel-like structure within the mass (**Fig. 1C**). 3D CT further identified an aberrant systemic artery, measuring up to 6 mm in diameter, arising from the abdominal aorta. Venous drainage from the lesion into the pulmonary vein was also suggested (**Fig. 2A**–**2E**). Additionally, the absence of the segmental arterial and bronchial branches expected to supply the involved basal region indicated that the lesion corresponded to a sequestered lung in the right lower lobe (**Fig. 2D** and **2F**). Based on these findings, the preoperative diagnosis was IPS of the right lower lobe (Pryce type III).

**Fig. 3:**
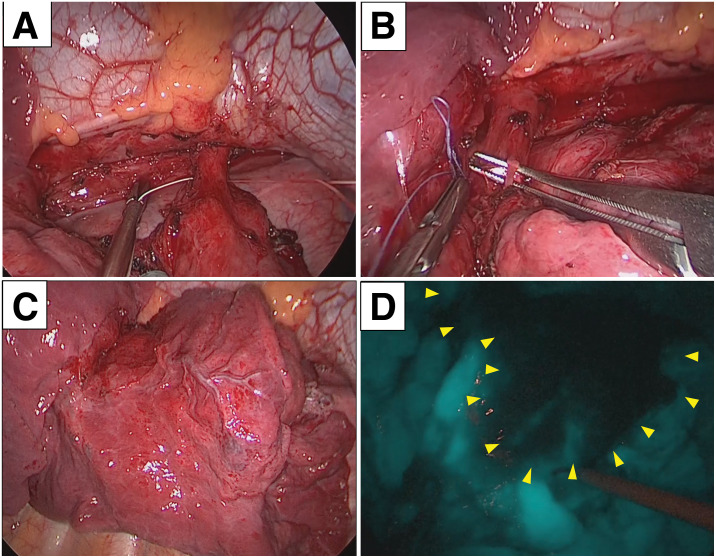
Intraoperative findings. (**A**) Proximal ligation and stapled division of the aberrant systemic artery. (**B**) Identification, ligation, and division of the basal pulmonary vein. (**C**) Thoracoscopic view before ICG administration, showing an indistinct boundary between normal lung and the sequestered area, making intersegmental demarcation difficult. (**D**) Intravenous ICG fluorescence clearly delineated the intersegmental plane (arrowheads) between perfused normal lung and the non-perfused sequestered area, enabling precise segmentectomy. ICG, indocyanine green

Three-port VATS was performed. Numerous string-like adhesions were observed in the thoracic cavity, likely because of chronic inflammation; the adhesions were mild and were easily lysed to obtain adequate exposure. During initial thoracoscopic inspection, the lung was inflated; however, adequate exposure could not be obtained, and the boundary between the normal lung and the sequestered area remained indistinct (**Fig. 3C**). The proximal portion of the aberrant systemic artery was first ligated with 2–0 silk (single ligation) and then divided using a stapler (**Fig. 3A**). Vascular stapling was performed with an ECHELON ENDOPATH 45-mm white reload (GST45W; Ethicon Endo-Surgery, Cincinnati, OH, USA). The pulmonary vein was dissected, and a venous branch draining the lesion into the basal pulmonary vein was identified, ligated, and divided (**Fig. 3B**). After a single 10-mg intravenous bolus of ICG (Daiichi Sankyo, Tokyo, Japan), fluorescence imaging clearly delineated the border between the normal lung and the sequestered area (**Fig. 3D**), allowing the right segmentectomy to be completed accordingly. ICG imaging was performed using a VISERA ELITE II (Olympus, Tokyo, Japan). The operation lasted 335 min, with 120 mL of intraoperative blood loss. The operative time was mainly influenced by additional intraoperative steps. These included thorough thoracic irrigation after minor leakage of cystic fluid during specimen retrieval and careful confirmation of complete cessation of an air leak from the remaining lung. No major intraoperative complications occurred, and the chest drain was removed on POD 1. The patient was discharged on POD 9. Follow-up CT at 6 months confirmed the absence of residual sequestered lung tissue.

**Fig. 4:**
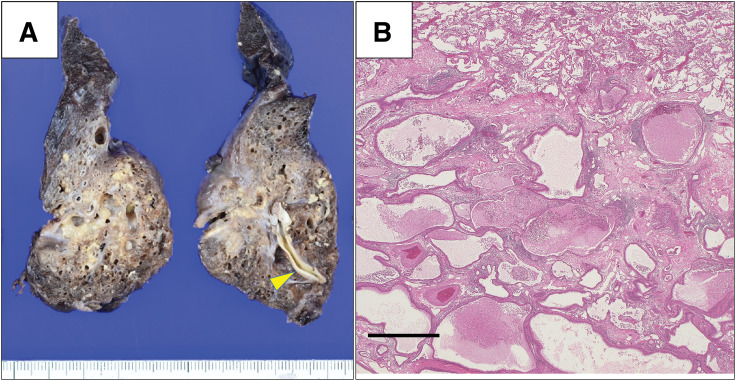
Gross and histopathology. (**A**) Gross photograph of the resected specimen (after formalin fixation) showing an aberrant systemic artery entering the lesion (arrowhead); the sequestered lung was removed with negative margins. (**B**) Hematoxylin and eosin staining demonstrating disruption of alveolar architecture with large cystic changes and neutrophil infiltration, consistent with chronic inflammation. Scale bar, 1 mm.

Gross examination revealed an aberrant systemic artery entering the lesion, and the sequestered lung was resected with negative margins (**Fig. 4A**). The aberrant artery had a thick muscular wall with features consistent with those of an elastic systemic artery. Histopathological evaluation with hematoxylin and eosin staining showed destruction of the alveolar architecture with large cystic changes and neutrophil infiltration consistent with chronic inflammation (**Fig. 4B**). No malignant findings or vasculitis were observed.

## DISCUSSION

The present case involved right lower lobe IPS (Pryce type III) in a patient with a history of recurrent pneumonia. Preoperative 3D-CT clearly delineated the aberrant systemic artery and venous drainage, and complete thoracoscopic lung-preserving (sublobar) resection was achieved after adhesiolysis for mild intrathoracic adhesions.

In sublobar resection for IPS, accurate identification of the resection line between normal lung and sequestrated lung is essential. Multiple techniques for ISP identification, including the inflation–deflation method, have been described.^2)^ The inflation–deflation method has been applied to IPS in prior case reports.^5,6)^ In Pryce type III IPS without bronchial communication, ventilation-based demarcation may theoretically be relatively clear because only the normal lung inflates; however, lung inflation itself can reduce the thoracoscopic working space and may compromise visualization, particularly when adhesions are present. In addition, severe pleural adhesions may limit the applicability of ventilation-based demarcation techniques.^2)^ Because pneumonia history can be associated with more extensive adhesions, an approach incorporating direct visualization (hybrid VATS) has also been selected in this setting.^6)^

ICG fluorescence, by contrast, visualizes perfusion differences rather than ventilation and can delineate the border without lung inflation. ICG-assisted sublobar resection for IPS has been reported in several case-based studies,^7–10)^ and representative reports—including the methods used to determine the resection line—are summarized in **Table 1**.^5–10)^

**Table 1:** Published case of IPS sublobar resections and resection-line identification methods

Author (year)	Age	Sex	History of pneumonia	Pryce type	Location	Approach	Intrathoracic adhesions	Feeding artery (origin, size)	Systemic artery management	ISP identification method	ICG dose (mg)	Time to fluorescence (s)	Operative time (min)	Blood loss (ml)	Postoperative hospital stay (days)	Complications
Shibuya et al. (2017)^5)^	6	Female	Yes	NR	LLL	hVATS (5 cm)	Mild–moderate	Descending aorta, NR	Ligation + clipping + energy device	Inflation–Deflation	NR	NR	217	56	5	No
Ose et al. (2018)^6)^	36	Female	No	III	LLL	cVATS	No	Abdominal aorta, NR	Double ligation	Inflation–Deflation	NR	NR	286	100	NR	No
Motohashi et al. (2020)^7)^	33	Male	Yes	III	LLL	cVATS	NR	Descending aorta, NR	Ligation + stapler	ICG	22.5 (in 3 divided doses)	NR	NR	NR	7	No
Petersen et al. (2023)^8)^	34	Female	Yes	NR	LLL	cVATS	Mild	Descending aorta, 10 mm	Stapler	ICG	5	NR	NR	NR	2	No
Kanno et al. (2023)^9)^	34	Female	No	III	RLL	cVATS	No	Celiac artery, 5 mm	Ligation + energy device	ICG	5	NR	NR	NR	NR	NR
Kim et al. (2023)^10)^	21	Female	No	NR	RLL	RATS	No	Descending aorta, NR	Stapler	ICG	10	NR	NR	NR	1	No
This case	21	Male	Yes	III	RLL	cVATS	Mild	Abdominal aorta, 6 mm	Ligation + stapler	ICG	10	8	335	120	9	No

cVATS, complete video-assisted thoracic surgery; hVATS, hybrid video-assisted thoracic surgery; ICG, indocyanine green; IPS, intralobar pulmonary sequestration; ISP, intersegmental plane; LLL, left lower lobe; NR, not reported; RATS, robotic-assisted thoracic surgery; RLL, right lower lobe

Careful patient selection remains crucial. Pryce classification is useful for anticipating perfusion patterns; in type III, the aberrant systemic artery supplies only the sequestrated lung, which may allow clearer demarcation once the feeding vessel is controlled.^11,12)^ The observation that many published lung-preserving resections have been performed in type III IPS supports this concept.^3,9)^ From a practical standpoint, we consider the following as key requirements when planning sublobar resection for IPS: (1) clear delineation of the aberrant systemic artery and venous drainage on 3D-CT, (2) an inflammatory/adhesive condition that permits safe dissection and reliable demarcation, and (3) patient factors (e.g., age and baseline pulmonary function) that justify the clinical benefit of lung preservation.^3,13)^ Minimally invasive approaches continue to expand, and in experienced centers, RATS may be a feasible alternative to open thoracotomy with potential perioperative recovery benefits.^10,14)^

A recognized limitation of ICG fluorescence is that inadequate visualization (“poor staining”) can occur in a subset of patients.^4)^ Reduced pulmonary vascular bed, including that associated with obstructive lung disease, has been reported as a factor related to suboptimal visualization,^4)^ and heavy smoking or emphysematous changes on CT may also decrease fluorescence contrast.^2)^ Importantly, in IPS, interpretation of fluorescence requires particular caution: after division of the feeding artery, a nonfluorescent area may represent either the sequestrated lung with true absence of perfusion or inadequate ICG delivery to normally perfused lung (“poor staining”). Conversely, if an unrecognized accessory systemic feeding vessel supplies part of the sequestrated tissue, that portion may fluoresce and be mistaken for normal lung, potentially leading to incomplete resection. Therefore, fluorescence findings should not be interpreted in isolation; the demarcation should be assessed in conjunction with preoperative 3D-CT vascular mapping (including careful assessment of the number and course of systemic feeding vessels and venous drainage), intraoperative anatomical landmarks (e.g., intersegmental veins), and the expected vascular territory after feeding-vessel control. In the present case, there was no obstructive lung disease and no apparent emphysematous change on imaging; thus, markedly inadequate visualization due to poor pulmonary perfusion was considered less likely. In addition, follow-up CT at 6 months demonstrated no residual sequestrated lung, which retrospectively supports the accuracy of the fluorescence-guided demarcation and resection line.

Accordingly, if ICG does not provide sufficient demarcation, the resection line should be determined using alternative approaches such as peripheral identification of anatomical landmarks (e.g., intersegmental veins) and/or methods reported for IPS including inflation–deflation.^2,5,6)^ If the resection line cannot be established with adequate confidence, prioritizing complete removal of sequestrated tissue is essential, and conversion to lobectomy is a reasonable option, particularly in cases with extensive inflammation or strong adhesions.^1,3,15)^

Management of the aberrant systemic artery is another critical issue because it carries systemic arterial pressure and may be of substantial caliber. Reported strategies include ligation, clipping, energy devices, and stapling.^16–18)^ Secure proximal control and meticulous handling are essential regardless of the technique.^16)^ In the present case, the artery was managed safely without vascular complications, and postoperative imaging showed no residual lesion and no aneurysm formation at the arterial stump; however, further accumulation of cases with standardized reporting of vascular control strategies and complications is warranted.

## CONCLUSIONS

In selected patients with IPS, ICG fluorescence can assist in identifying the resection line even in the presence of intrathoracic adhesions and may facilitate thoracoscopic lung-preserving resection. Careful patient selection with a contingency strategy for inadequate demarcation is essential, and lobectomy should be considered when reliable demarcation or safe dissection cannot be ensured.
